# Influence of probiotics and deoxycholate on azathioprine transport in the PAMPA model: insights into pharmacomicrobiomics and interindividual variability in drug response

**DOI:** 10.3389/fphar.2025.1608110

**Published:** 2025-06-26

**Authors:** Maja Đanić, Nebojša Pavlović, Natalija Dedić, Dragana Zaklan, Slavica Lazarević, Bojan Stanimirov, Momir Mikov

**Affiliations:** ^1^ Department of Pharmacology, Toxicology and Clinical Pharmacology, Faculty of Medicine, University of Novi Sad, Novi Sad, Serbia; ^2^ Department of Pharmacy, Faculty of Medicine, University of Novi Sad, Novi Sad, Serbia; ^3^ Faculty of Medicine, University of Novi Sad, Novi Sad, Serbia; ^4^ Department of Biochemistry, Faculty of Medicine, University of Novi Sad, Novi Sad, Serbia

**Keywords:** drug transport, probiotics, permeability, intestinal microbiota, azathioprine, inflammatory bowel disease, drug optimization

## Abstract

**Introduction:**

Interindividual variability in drug response presents a major clinical challenge, necessitating a deeper understanding of contributing factors. While the role of gut microbiota, probiotics and bile acids in modulating drug metabolism, absorption, and bioavailability is increasingly recognized, their precise impact on variability remains an active area of research. Azathioprine, a widely used immunosuppressant for inflammatory bowel disease, exhibits significant variability in patient response. This study investigates the effects of probiotic bacteria and sodium deoxycholate (DC) on azathioprine permeability to elucidate mechanisms underlying interindividual differences in drug absorption and therapeutic outcomes.

**Methods:**

The parallel artificial membrane permeability assay (PAMPA) was used to evaluate the permeability of azathioprine at pH 5.8, 6.5, and 7.4, both alone and in combination with DC and probiotics. Following a six-hour incubation, azathioprine concentrations were quantified using high-performance liquid chromatography (HPLC), and permeability coefficients were calculated. Additionally, molecular mechanics (MM2) calculations were performed to analyze interactions between azathioprine and bile acids. Chemoinformatics-based platforms, pkCSM and ADMETsar, were used to predict the interactions of azathioprine and DC with drug transporters in the gastrointestinal tract, particularly P-glycoprotein (P-gp).

**Results:**

Azathioprine exhibited higher permeability at lower pH values. The presence of probiotic bacteria resulted in a statistically significant increase in azathioprine permeability; however, the total amount of azathioprine during incubation with bacteria significantly decreased. DC reduced drug permeability, with higher DC concentrations leading to a greater decrease in azathioprine permeability, as reflected by lower drug levels in the acceptor compartment, likely due to the formation of hydrophilic complexes with azathioprine, which exhibit lower membrane permeability compared to the free drug. *In silico* analysis suggested that azathioprine absorption may involve intestinal transport proteins, including P-gp, and that DC, as a P-gp inhibitor, could additionally affect its absorption and bioavailability through this mechanism.

**Conclusion:**

The findings indicate significant interactions between probiotic bacteria, DC, and azathioprine that may affect azathioprine absorption. Since the PAMPA method is exclusively suited for evaluating passive transport, additional *in vitro* and *in vivo* studies are required to further investigate the interactions of azathioprine with intestinal bacteria and bile acids, ultimately determining their impact on intestinal absorption and bioavailability.

## 1 Introduction

In recent years, pharmacomicrobiomics has emerged as a cutting-edge discipline dedicated to exploring the intricate interactions between gut microbiota and drugs. The primary focus of pharmacomicrobiomics is to understand how variations in the gut microbiota contribute to inter-individual differences in drug responses (Dikeocha et al., 2022). A remarkable metabolic capacity of gut microbiota and its unique microbial fingerprint in each individual are increasingly recognized as major contributors to variability observed in drug response. Drugs particularly susceptible to the influence of gut microbiota include those with low solubility and/or permeability or those formulated for modified release, as their prolonged residence time in the gastrointestinal tract allows extensive interactions with intestinal bacteria (Stojančević et al., 2014; Enright et al., 2016; Zhao et al., 2023). Intestinal bacteria harbor a broad spectrum of enzymes capable of catalyzing diverse biotransformation reactions, such as reduction, hydrolysis, acetylation, deamination, dehydroxylation, decarboxylation, demethylation, deconjugation, and proteolysis (Thiele et al., 2017). Beyond enzymatic biotransformation, recent evidence highlights the ability of gut bacteria to modulate drug bioavailability and efficacy through bioaccumulation, which refers to the ability of bacteria to store certain drugs intracellularly (Klünemann et al., 2021; Đanić et al., 2023). For instance, drugs like simvastatin (Đanić et al., 2023), gliclazide (Ðanić et al., 2019) and duloxetine (Klünemann et al., 2021) have been shown to accumulate in gut bacteria, potentially altering their pharmacokinetics and therapeutic effects. This dual capacity of gut bacteria to metabolize and sequester drugs underscores their profound impact on drug disposition and therapeutic outcomes. Recent studies have expanded the scope of pharmacomicrobiomics to include bidirectional interactions, emphasizing also the impact of drugs on gut microbiota function and compositions and its effect on therapeutic responses and patient prognosis. This broadened perspective has led to the proposal of the term “pharmacoecology” to describe the dynamic interplay between drugs and the gut microbial ecosystem (Heirali et al., 2023). By unraveling these complex interactions, pharmacomicrobiomics offers a pathway to optimize drug dosing, minimize adverse effects, and personalize therapeutic strategies based on an individual’s microbiome profile (ElRakaiby et al., 2014; Scher et al., 2020; Lazarević et al., 2022).

Over the past decade, the pivotal role of gut microbiota has been increasingly recognized in numerous immune-mediated diseases, including inflammatory bowel disease (IBD) (Lane et al., 2017). This has fueled growing interest in understanding the complex interactions between gut microbiota and therapeutic agents used in the management of IBD. Among these therapies, azathioprine has long been considered a cornerstone treatment due to its efficacy in maintaining remission and reducing disease progression. However, despite its widespread use, the therapeutic response to azathioprine exhibits significant interindividual variability, presenting a major challenge in clinical practice and underscoring the importance of personalized treatment strategies (Lazarević et al., 2022). Azathioprine is a prodrug that undergoes a series of enzymatic conversions after oral administration to produce its active metabolites, including 6-thioguanine nucleotides (6-TGNs), which are responsible for its immunosuppressive effects. According to the Biopharmaceutics Classification System (BCS), azathioprine belongs to Class IV, characterized by low solubility and low permeability, which poses challenges in developing formulations with optimal oral bioavailability (Bocci et al., 2022). Azathioprine absorption is thought to occur through a combination of passive diffusion and active transport (Karbelkar and Majumdar, 2016). While specific carrier-mediated uptake mechanisms for azathioprine are not well-defined, its metabolite 6-mercaptopurine (6-MP) may interact with nucleoside transporters. Although azathioprine is not strongly recognized as a substrate for P-glycoprotein (P-gp), which acts as an efflux transporter that may reduce intestinal drug absorption by pumping the drug back into the intestinal lumen (DuBuske, 2005), genetic polymorphisms in the *MDR1* gene, which encodes P-gp, have been associated with interindividual variability in azathioprine absorption and therapeutic response (Rosso et al., 2009). Additionally, Karbelkar et al. demonstrated that azathioprine is a potential substrate for peptide transporters such as peptide pransporter-1 (PEPT1). Their study showed that methotrexate-induced intestinal mucositis reduced the systemic bioavailability of azathioprine, which was attributed to decreased intestinal absorption of azathioprine due to lower expression of PEPT1 in the affected intestinal mucosa (Karbelkar and Majumdar, 2016). The bioavailability of azathioprine has been reported to vary substantially, ranging from 27% to 83% (Van Os et al., 1996). Similarly, the levels of 6-TGNs, which are critical for therapeutic efficacy, show marked interindividual variability, with concentrations ranging from undetectable to as high as 413 pmol per 8 × 10^8^ red blood cells (Chan et al., 1990). These disparities are clinically significant, as subtherapeutic levels are associated with treatment failure, while excessive levels increase the risk of adverse effects such as myelosuppression. While genetic polymorphisms in key enzymes such as thiopurine S-methyltransferase (TPMT) have been well-documented as contributors to variability in thiopurine metabolism (Heckmann et al., 2005), emerging evidence suggests that the gut microbiota may also play a critical role. Recent studies have suggested that certain bacterial species possess enzymatic capabilities to directly metabolize thiopurines, potentially altering their pharmacokinetic profiles and therapeutic outcomes (Lazarević et al., 2022).

The primary aim of our study was to develop and apply a fast and reliable method for the preliminary assessment of microbiota–azathioprine interactions in the context of pharmacomicrobiomics, with a specific focus on the influence of gut microbiota on azathioprine transport and metabolism. To this end, probiotic bacteria from a commercially available product were used as a simplified model. Probiotics were selected for two main reasons: first, they represent a natural and beneficial component of the gut microbiota; and second, they are readily available in standardized formulations containing well-characterized, viable bacterial strains. These properties make probiotics a practical and relevant model for preliminary evaluations of microbiota–drug interactions, as we have already demonstrated in our previous studies involving other drugs such as simvastatin (Đanić et al., 2023) and gliclazide (Ðanić et al., 2019).

Considering the ability of probiotics to modulate the composition and activity of the gut microbiota, they have been increasingly investigated for their potential role in both the prevention and treatment of IBD. Their capacity to reshape microbial communities, enhance intestinal barrier function, regulate local immune responses, and potentially influence drug metabolic pathways makes them promising candidates as adjuncts to conventional therapeutic regimens (Roy and Dhaneshwar, 2023; Wang et al., 2024). The use of probiotics represents a simple, safe and cost-effective approach that may contribute to disease control, induction of remission, prevention of relapse, reduction of inflammation and overall improvement in the quality of life of patients with IBD This underscores the importance of investigating the combined use of probiotics alongside established therapies to maximize therapeutic efficacy. A thorough understanding of the interactions between probiotics and conventional drugs is crucial for optimizing treatment outcomes and minimizing potential adverse effects, thereby advancing personalized approaches in IBD management.

In addition to gut microbiota and probiotics, bile acids, which are also physiologically present in the intestinal tract, may interact with drugs, affecting their absorption and bioavailability. At concentrations above the critical micellar concentration (CMC), bile acids can increase the solubility and dissolution rate of lipophilic drugs. These biomolecules can increase drug bioavailability even at submicellar levels by improving the solubility and dissolution rate of lipophilic drugs, as well as by promoting partitioning into cell membranes, thereby enhancing membrane fluidity and permeability. Additionally, bile acids may affect transporter-mediated absorption of both physically complexed and chemically conjugated drug molecules (Mooranian et al., 2021; Pavlović et al., 2018; Djanic et al., 2016; Stojančević et al., 2013). The final outcome of bile acids’ influence on drug transport through biological membranes depends on various factors, including the type and structure of bile acids, their hydrophobicity, and concentration (Stojančević et al., 2013).

To shed more light on these interactions between azathioprine, bile acids, and probiotic bacteria at intestinal level and uncover novel insights into interindividual differences in thiopurine therapy, the aim of our study was to elucidate the underlying mechanisms under *in vitro* conditions, with a particular focus on transport processes, simulating absorption and the gastrointestinal environment.

## 2 Methods

### 2.1 Chemicals

Azathioprine and sodium deoxycholate (DC) were purchased from Sigma Chemicals Co, St. Louis, MO, USA. Commercial probiotic capsules (PROBIOTIC^®^, Hemofarm AD, Serbia), containing ≥5 × 10^9^ colony-forming units (CFU) of lyophilized probiotic strains *Lactobacillus helveticus* Rosell-52 (formerly *Lactobacillus acidophilus* Rosell-52), *Lactobacillus rhamnosus* Rosell-11, and *Bifidobacterium longum* Rosell-175 per capsule, were obtained from Hemofarm AD, Serbia.

Phosphate buffered saline (PBS) pH 7.4 was purchased from Gibco, Life Technologies, Grand Island, NY, USA. Lecithin and dodecane were obtained from Carl Roth, Germany. Buffers with pH values of 6.5 and 5.8 were prepared by adjusting the pH of PBS 7.4 using HCl to the desired pH values. Water, acetonitrile, dimethyl sulfoxide (DMSO), orthophosphoric acid and triethylamine were of HPLC grade (J.T. Baker, Phillipsburg, NJ, USA). Lecithin and dodecane were obtained from Carl Roth, Germany.

### 2.2 Preparation of solutions

The stock solution of azathioprine (5 mg/mL) was prepared by dissolving in DMSO. The stock solution was then diluted 100 times with the appropriate buffer to achieve a final concentration of 50 μg/mL. Three pH values were used in the study: pH 5.8, 6.5 (adjusted by adding hydrochloric acid to PBS 7.4), and 7.4. The selected pH values were intended to simulate the physiological conditions of the gastrointestinal tract, from the proximal small intestine to the distal ileum and colon, where gut microbiota is most abundant, in order to reflect the potential absorption sites of azathioprine following oral administration.

The standard azathioprine solutions for the calibration curve were prepared by diluting the stock solution with the mobile phase to final concentrations ranging from 0.038 to 10 μg/mL. The dependence of the area under the curve on the concentration was analyzed, and the correlation coefficient of the calibration curve was *R* ^2^ = 0.9999. The equation of the calibration curve was y = 2.1193x + 0.0186.

The stock solution of DC (2.5 mM, equivalent to 1.037 mg/mL) was prepared by dissolving the salt in water. In this study, submicellar concentrations of DC (0.25 mM, 0.125 mM, 0.0625 mM) were used. In the donor compartments of the groups with probiotic bacteria, an appropriate amount of the probiotic capsule content was added to achieve a concentration of 5 × 10^8^ CFU/mL.

### 2.3 Experimental groups

To investigate the effects of probiotic bacteria and DC on the transport of azathioprine, the following groups were formed (for each pH value).

• A–azathioprine solution (25 μg/mL) in respective buffer
• AD1 – azathioprine solution (25 μg/mL) with the addition of DC (0.0625 mM)
• AD2 – azathioprine solution (25 μg/mL) with the addition of DC (0.125 mM)
• AD3 – azathioprine solution (25 μg/mL) with the addition of DC (0.25 mM)
• AP–probiotic bacteria (5 × 10^8^ CFU/mL) in azathioprine solution (25 μg/mL)
• AD1P–probiotic bacteria (5 × 10^8^ CFU/mL) in azathioprine solution (25 μg/mL) with the addition of DC (0.0625 mM)
• AD2P–probiotic bacteria (5 × 10^8^ CFU/mL) in azathioprine solution (25 μg/mL) with the addition of DC (0.125 mM)
• AD3P–probiotic bacteria (5 × 10^8^ CFU/mL) in azathioprine solution (25 μg/mL) with the addition of DC (0.25 mM)

The entire experiment was conducted simultaneously at three different pH values (pH 5.8, pH 6.5, and pH 7.4) and repeated in triplicate.

### 2.4 PAMPA permeability test

A Parallel Artificial Membrane Permeability Assay (PAMPA) was applied to investigate the permeability of azathioprine, both alone and in combination with DC and probiotics, according to the previously published method (Đanić et al., 2021; Lazarević et al., 2025). Hydrophobic MultiScreen PVDF microfiltration plates with 96 wells and a pore diameter of 0.45 μm (Millipore, USA) were used as acceptor plates and as supports for the artificial membrane. Each well of the acceptor plate was impregnated with 6 μL of a 2% lecithin solution in n-dodecane, and after solvent evaporation, an artificial membrane was formed. To the corresponding wells of the acceptor plate, 200 μL of PBS buffer (pH 7.4) was added, while 300 μL of the tested compound solution in respective buffer was added to the wells of the donor plate (MultiScreen Transport Receiver Plate, Millipore, USA). All analyses were conducted in triplicate at three different pH values in the donor compartment, while the pH value in the acceptor compartment was always 7.4. The donor plate was aligned with the acceptor plate to initiate incubation. Incubation of samples was performed at 37°C in a sealed PAMPA system to minimize oxygen exposure. After incubation for 6 h with constant gentle mixing, the plates were separated, and sampling was performed from each donor and acceptor well to determine the azathioprine concentration in both compartments using the HPLC method. Before HPLC injection, protein precipitation was achieved by diluting a 50 μL sample from the donor compartment fivefold with acetonitrile, followed by centrifugation at 15,000 rpm for 10 min at +4°C, as described by Đanić et al. (Ðanić et al., 2019). Subsequently, 100 μL of the supernatant was directly injected into the HPLC system. To assess the effect of probiotic bacteria on the total azathioprine mass, considering potential accumulation or biotransformation, the total mass was calculated as the sum of the mass in both the acceptor and donor compartments.

After determining the concentrations of the tested compounds in the donor and acceptor wells, apparent permeability coefficients (P_app_) were calculated and expressed in units of cm/s based on the equation:

$$Papp=C\times \left[-\ln\;\left(1-\frac{Ca}{Ceq}\right)\right]$$

where *C* is a correction factor calculated as:

$$C=\frac{Vd\times Va}{\left(Vd+Va\right)\times S\times t}$$

The equilibrium drug concentration (*Ceq*) was determined using mass balance:

$$Ceq=\frac{Cd\times Vd+Ca\times Va}{Vd+Va}$$

**FIGURE 1:**
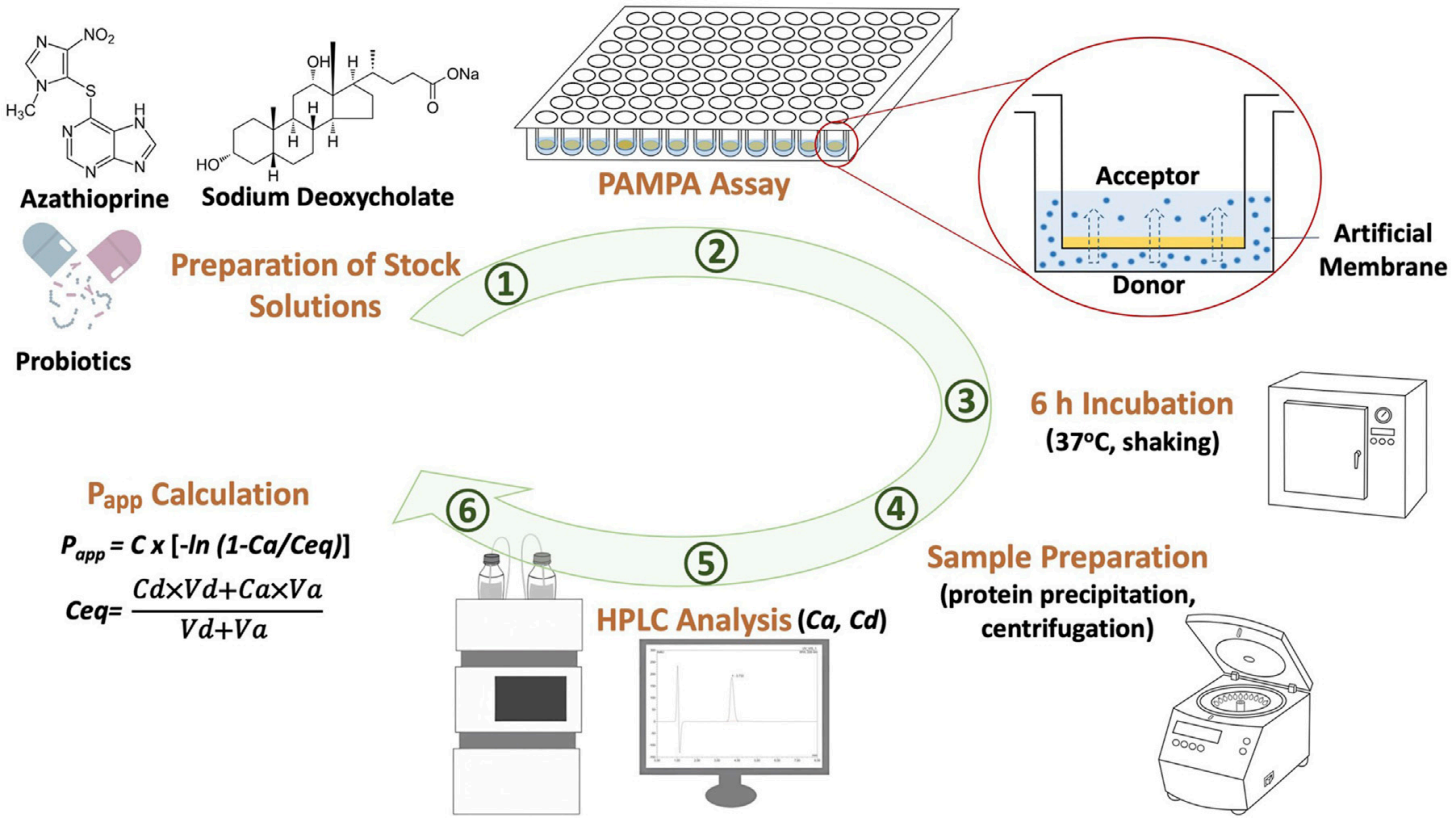
Graphical summary of the experimental steps of the study.

In these equations, *Ca* represents the drug concentration in the acceptor compartment after 6 h, *Ceq* is the equilibrium drug concentration determined based on mass balance, *Va* and *Vd* denote the volumes of the acceptor and donor compartments, respectively, measured in milliliters, *t* represents the time period over which permeability was assessed, expressed in seconds, and *S* refers to the membrane surface area in square centimeters. The surface area of the artificial membrane was calculated to be 0.24 cm^2^, as the acceptor filter membrane plates have a surface area of 0.32 cm^2^ and a porosity of 75%, according to the manufacturer’s data. A schematic overview of the research workflow is shown in Figure 1.

### 2.5 HPLC analysis

Sample analysis was performed using high-performance liquid chromatography (HPLC; Dionex) with a diode array detector (DAD). The concentration of azathioprine was determined according to a previously published method (Ravisankar et al., 2015) with slight modifications. Briefly, the chromatographic analysis was performed using a reverse-phase Zorbax Eclipse Plus C18 column (150 mm × 2.1 mm, 5 μm; Agilent Technologies, USA), coupled with a Zorbax Extend C18 guard column (12.5 mm × 2.1 mm, 5 μm; Agilent Technologies, USA). During the analysis, the column temperature was maintained at a constant 25°C, and the injection volume was 20 μL. Elution was performed using an isocratic program, with the mobile phase consisting of 50% AcN and 50% phosphate buffer adjusted to pH 3.3 with orthophosphoric acid, with the addition of 0.5 mL triethylamine as a column modifier. The total analysis time was 5 min, with a constant flow rate of the mobile phase at 0.3 mL/min. The retention time of azathioprine was 2.1 min. The eluate was monitored using a UV/DAD detector at a wavelength of 276 nm.

### 2.6 Structural modeling and geometric optimization

The initial 3D structures of azathioprine and DC were prepared using Perkin Elmer Chem3D (version 18.0.0.231) software. The initial 3D structure of their complex was constructed in the same manner. Molecular geometries were optimized using molecular mechanics calculations (MM2), as implemented in the Chem3D software.

### 2.7 *In silico* analysis

To predict the interaction profiles of azathioprine and DC with P-gp, *in silico analysis* was performed using the publicly available chemoinformatics-based platforms, pkCSM (Pires et al., 2015) and ADMETsar (Cheng et al., 2012). In addition to predicting various pharmacokinetic properties using machine learning modelling, the pkCSM software predicts a binary outcome indicating whether a compound is likely to be a substrate and/or an inhibitor for P-gp. On the other hand, ADMETsar provides a probabilistic output for the compound’s likelihood of being a substrate or an inhibitor of P-gp. A value closer to 1 indicates a higher probability of the compound being a substrate or inhibitor, while a value closer to 0 suggests a lower likelihood.

### 2.8 Statistics

Statistical analysis was conducted using IBM SPSS Statistics software, version 21. All analyses were performed in triplicate. The results are expressed as the arithmetic mean ± standard deviation (SD). The statistical significance of differences between mean parameter values was assessed using one-way analysis of variance (ANOVA) followed by Tukey’s post-hoc test for multiple comparisons. Statistical hypotheses were tested at a significance level of 5% (p < 0.05).

## 3 Results

### 3.1 Azathioprine concentrations in the acceptor compartment

**FIGURE 2:**
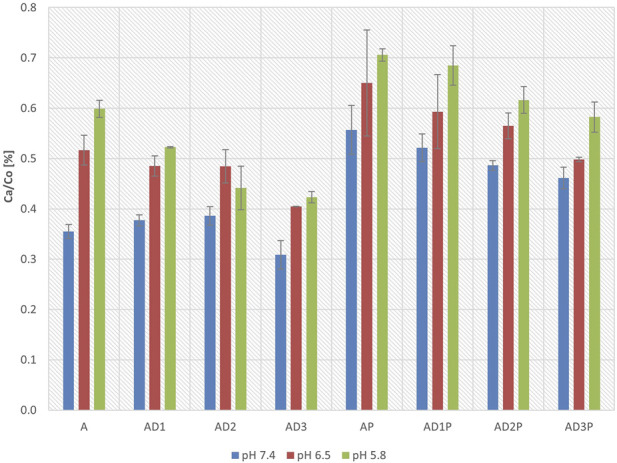
Azathioprine concentrations in the acceptor compartment after 6 h of incubation relative to initial concentrations in the donor compartment at pH 7.4, 6.5, and 5.8, expressed as percentages.

Figure 2 presents the concentrations of azathioprine in the acceptor compartment after 6 h of incubation, expressed as a percentage of the initial azathioprine concentrations in the donor compartment at three different pH values.

In all tested groups, an increase in azathioprine concentration in the acceptor compartment was observed as the pH decreased, indicating higher drug permeability in a more acidic environment.

At all three pH values, the groups in which azathioprine was incubated with probiotics exhibited higher drug concentrations in the acceptor compartment compared to the control groups without probiotics (AP vs. A). The most pronounced increase (57%) was observed at pH 7.4 (AP vs. A, p = 7.27 × 10^−7^), whereas at pH 5.8, the increase was smaller, reaching 18% (AP vs. A, p = 0.003). At pH 6.5, azathioprine concentrations were also higher; however, the differences were not statistically significant. Similarly, in the DC-containing groups, probiotic co-incubation led to increased azathioprine concentrations across all three pH values, with statistically significant differences observed at pH 7.4 (AD1P vs. AD1, p = 6.51 × 10^−5^; AD2P vs. AD2, p = 0.003; AD3P vs. AD3, p = 3.2 × 10^−5^) and pH 5.8 (AD1P vs. AD1, p = 2.61 × 10^−5^; AD2P vs. AD2, p = 1.03 × 10^−5^; AD3P vs. AD3, p = 3.27 × 10^−5^), while at pH 6.5, the differences did not reach statistical significance.

Furthermore, groups containing DC exhibited lower azathioprine concentrations in the acceptor compartment compared to groups without DC. At pH 5.8, significantly lower concentrations were recorded in all DC-containing groups compared to the control groups (AD1 vs. A, p = 0.04; AD2 vs. A, p = 3.9 × 10^−5^; AD3 vs. A, p = 9.5 × 10^−6^), while in probiotic containing groups, statistical significance was observed at intermediate and high DC concentrations (AP vs. AD2P, p = 0.013; AP vs. AD3P, p = 6.33 × 10^−4^). Statistically significant differences were observed with DC in probiotic groups at pH 7.4 (AD3P vs. AP, p = 0.005) and pH 6.5 (AD3P vs. AP, p = 0.029), with the lowest azathioprine concentrations found at the highest DC concentration.

When comparing the effects of different DC concentrations, at pH 7.4 and 6.5, despite a decrease in azathioprine concentration in the acceptor compartment with increasing DC concentrations, statistical significance was not achieved. However, at pH 5.8, groups with intermediate and high DC concentrations showed significantly lower azathioprine concentrations in the acceptor compartment compared to the group with the lowest DC concentration (AD1 vs. AD2, p = 0.030; AD1 vs. AD3, p = 0.005). In probiotic-treated groups, a significantly lower azathioprine concentration was observed in the group with the highest DC concentration compared to the group with the lowest DC concentration (AD3P vs. AD1P, p = 0.004).

### 3.2 Azathioprine concentrations in the donor compartment

**FIGURE 3:**
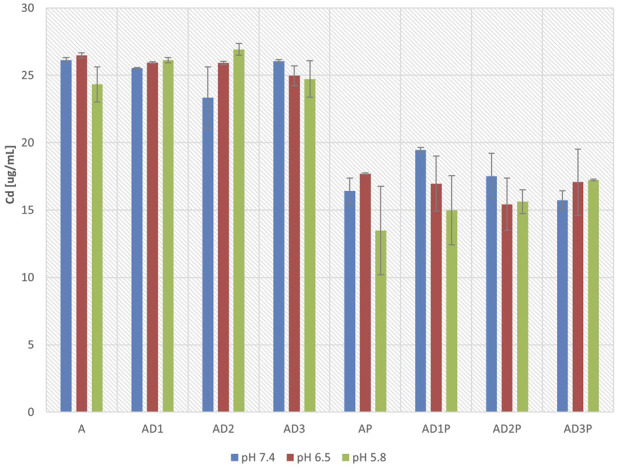
Azathioprine concentrations in the donor compartment after 6 h of incubation at pH 7.4, 6.5, and 5.8.

Figure 3 presents the concentrations of azathioprine in the donor compartment of the PAMPA membrane after 6 h of incubation. The concentrations were measured at pH 7.4, 6.5, and 5.8.

In general, no statistically significant differences were observed in azathioprine concentrations within the same groups at different pH values. In the donor compartment, a statistically significant decrease in azathioprine concentration was observed in all groups containing probiotic bacteria, with a reduction of up to 45% at pH 5.8 (AP vs. A, p = 1.2 × 10^−5^), 33% at pH 6.5 (AP vs. A, p = 1.35 × 10^−5^), and 37% at pH 7.4 (AP vs. A, p = 1.32 × 10^−7^). Statistically significant reductions in azathioprine concentrations were also observed in the groups containing probiotics and DC, compared to the corresponding control groups without probiotics.

The co-incubation of azathioprine with DC did not lead to statistically significant changes in azathioprine concentrations in the donor compartment, when compared to the groups without DC, across all three pH values.

When assessing the impact of varying DC concentrations, at pH 7.4, in the presence of probiotics, a statistically significantly lower azathioprine concentration in the donor compartment was observed in the group with the highest DC concentration compared to the group with the lowest DC concentration (AD1P vs. AD3P, p = 0.012). However, in the remaining groups at pH 7.4, as well as in all tested groups at pH 6.5 and 5.8, no statistically significant difference in azathioprine concentration in the donor compartment was observed when analyzing the concentration-dependent effect of DC in any of the tested groups.

### 3.3 The total mass of azathioprine after 6 hours of incubation

**FIGURE 4:**
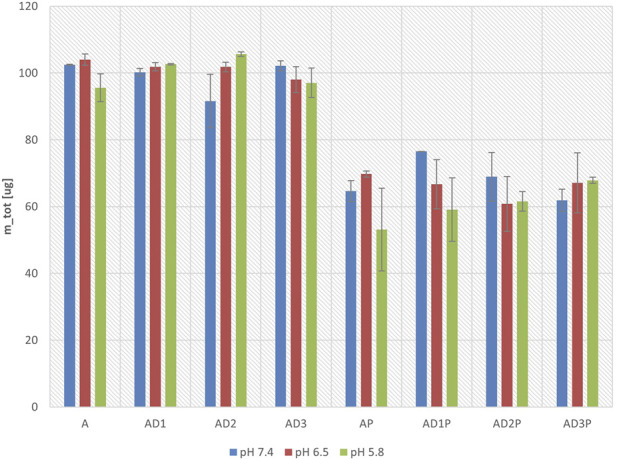
Total mass of azathioprine after 6 hours of incubation at pH 7.4, 6.5, and 5.8, expressed as a percentage of the initial mass.

Figure 4 illustrates the change in the total mass of azathioprine before and after 6 h of incubation at pH 7.4, 6.5, and 5.8. The total mass before and after incubation was calculated as the sum of the masses in the acceptor and donor compartments. The change in total mass is expressed as a percentage of the initial mass before incubation. It can be observed that at all the observed pH values, there is a statistically significant reduction in the total mass in the probiotic groups, reaching approximately 60% of the initial value when considering all probiotic groups together.

### 3.4 Azathioprine permeability

Table 1 provides an overview of the permeability of azathioprine across different pH values (7.4, 6.5, and 5.8). Permeability is expressed through the apparent permeability coefficient (*P*_*app*_). Generally, values of the coefficient less than 1 × 10^−6^ cm/s indicate poor permeability, while values greater than this threshold suggest good permeability.

**TABLE 1:** Permeability of azathioprine at pH 7.4, 6.5, and 5.8.

Groups	Permeability [P_app_ x10^−7^] [cm/s]		
	pH 7.4	pH 6.5	pH 5.8
A	1.34 ± 0.05	1.93 ± 0.14	2.43 ± 0.18
AD1	1.46 ± 0.02	1.84 ± 0.05	1.97 ± 0.0006
AD2	1.65 ± 0.22	1.84 ± 0.10	1.62 ± 0.17
AD3	1.17 ± 0.12	1.60 ± 0.06	1.69 ± 0.12
AP	3.34 ± 0.13	3.62 ± 0.54	5.37 ± 1.21
AD1P	2.64 ± 0.14	3.52 ± 0.83	4.62 ± 1.02
AD2P	2.76 ± 0.24	3.64 ± 0.33	3.89 ± 0.02
AD3P	2.90 ± 0.29	2.92 ± 0.43	3.33 ± 0.13

In the majority of groups, it is observed that azathioprine exhibits higher permeability at lower pH levels, with the lowest permeability observed at pH 7.4.

Regarding the impact of probiotic bacteria on azathioprine permeability, groups containing probiotics (AP, AD1P, AD2P, and AD3P) exhibit significantly higher permeability compared to control groups (A, AD1, AD2, AD3) at all three pH values.

Furthermore, the effect of sodium deoxycholate (DC) in combination with probiotics leads to a reduction in azathioprine permeability. Statistically significant reductions in permeability were noted in specific groups: AD1P vs. AP at pH 7.4 (p = 0.003), AD2P vs. AP at pH 7.4 (p = 0.015), and AD3P vs. AP at pH 5.8 (p = 0.0087). The concentration-dependent effect of DC was most pronounced at pH 5.8, where an increase in DC concentration led to a decrease in drug permeability.

### 3.5 Molecular mechanics calculations of the interaction between azathioprine and deoxycholic acid

Table 2 shows the minimized energies of azathioprine, deoxycholic acid (DCA), and their complex. The minimized total energies of azathioprine and DCA were 158.66 kcal/mol and 55.21 kcal/mol, respectively. The total energy of the azathioprine-DCA complex (196.93 kcal/mol) was lower than the sum of the potential energies of the two individual compounds optimized through molecular mechanics calculations, indicating that the formation of the complex resulted in system stabilization. Energy decomposition revealed that stretching (4.48 kcal/mol), bending (170.02 kcal/mol), stretch–bend (0.46 kcal/mol), and torsional (23.21 kcal/mol) terms contributed to the bonded interactions in the complex. Nonbonded contributions included non-1,4 van der Waals (VDW) (−29.04 kcal/mol), 1,4 VDW (30.89 kcal/mol) and dipole–dipole (−3.09 kcal/mol) interactions. The negative values for non-1,4 VDW and dipole–dipole indicate their stabilizing effect on the complex.

**TABLE 2:** Minimized total energy of azathioprine, DCA, and their complex.

E_AZA_ (kcal/mol)	E_DCA_ (kcal/mol)	E_AZA+_E_DCA_ (kcal/mol)	E_COMPLEX_ (kcal/mol)	ΔE (kcal/mol)
158.66	55.21	213.87	196.93	−16.94

**FIGURE 5:**
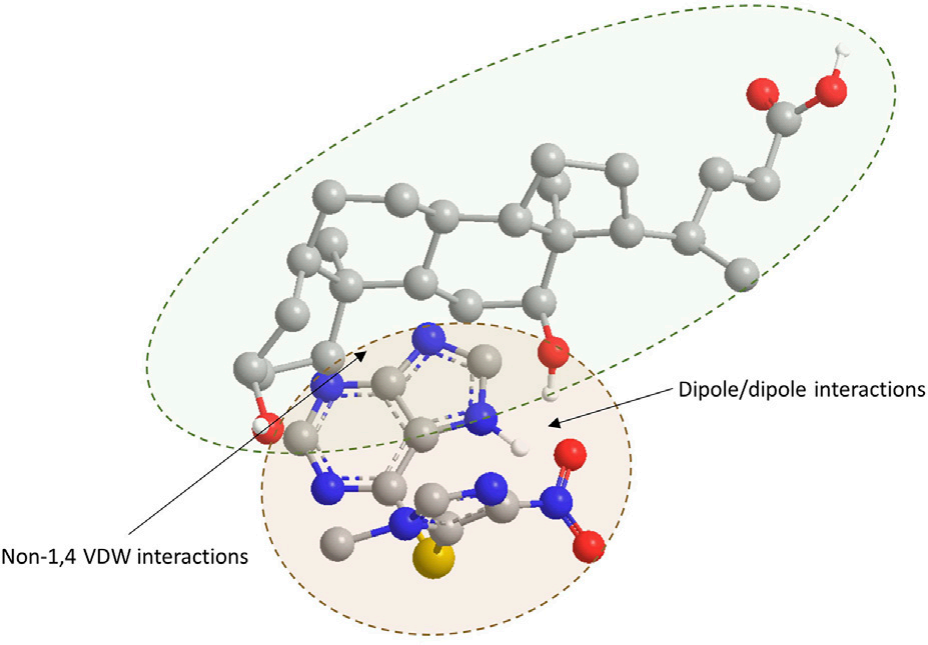
Geometrically optimized three-dimensional structure of the azathioprine/DCA complex. Atoms are color-coded as follows: oxygen (red), nitrogen (blue), sulfur (yellow), carbon (grey), and polar hydrogen (white). For clarity, hydrogen atoms bonded to carbon are omitted. The structure illustrates key intermolecular interactions stabilizing the complex.

As shown in Figure 5, which represents the geometrically optimized three-dimensional structure of the azathioprine/DC complex, the primary stabilization of the azathioprine/DCA complex arises from non-1,4 VDW interactions and attractive dipole/dipole forces between the two molecules.

### 3.6 *In silico* prediction of azathioprine and deoxycholate as substrates and/or inhibitors of P-glycoprotein

Table 3 presents the predicted interactions of azathioprine and DC with P-gp and its isoforms, P-gp1 and P-gp2, based on the results from pkCSM and ADMETsar software tools. According to pkCSM, azathioprine is predicted to be a P-gp substrate, while DC is not. Neither compound is predicted to be an inhibitor of P-gp1, but DC is identified as a P-gp2 inhibitor, in contrast to azathioprine, which is not predicted to inhibit P-gp2.

**TABLE 3:** Predicted interactions of azathioprine and sodium deoxycholate with P-glycoprotein and its isoforms, P-gp1 and P-gp2, based on *in silico* analysis.

Prediction	Azathioprine	Sodium deoxycholate	Unit
pkCSM software			
P-gp Substrate	Yes	No	Categorical (Yes/No)
P-gp1 Inhibitor	No	No	Categorical (Yes/No)
P-gp2 Inhibitor	No	Yes	Categorical (Yes/No)
ADMETsar software			
P-gp Substrate	0.153	0.285	Probability
P-gp Inhibitor	0.080	0.642	Probability

pkCSM software classifies the results into two categories: Yes or No, indicating whether the compound is predicted to be a P-gp substrate or inhibitor. ADMETsar, software provides probability values indicating the likelihood of the compound being a P-gp substrate or inhibitor, where values closer to 0 suggest low probability, 0.3 to 0.7 indicate moderate probability, and values closer to 1 represent high probability.

The ADMETsar software, which provides probability values, gives a lower probability for azathioprine being a P-gp substrate (0.153) and a very low probability of being a P-gp1 inhibitor (0.080). On the other hand, DC has a higher likelihood of interacting with P-gp1 as an inhibitor (0.642), but the probability of it being a P-gp substrate is also low (0.285).

## 4 Discussion

Our study focused on the largely unexplored but highly significant interactions between azathioprine, probiotic bacteria, and the bile acid representative DC, given their potential impact on the pharmacokinetics of azathioprine and their role in influencing therapeutic outcomes in patients. To gain insight into the mechanisms of these interactions at the intestinal absorption level, the PAMPA model was used, an established *in vitro* system for evaluating passive permeability across an artificial lipid membrane. Given that passive diffusion is the predominant mechanism of drug absorption in the gastrointestinal tract, with over 95% of known drugs relying on this process, the PAMPA model serves as a valuable tool for assessing drug absorption and bioavailability of orally administered drugs (Wu et al., 2011).

Given that pH variations in the gastrointestinal tract can influence drug ionization and permeability, our study was conducted at pH values of 5.8, 6.5, and 7.4 to assess how these changes affect the permeability of azathioprine. Azathioprine is a weak acid with a pKa of 7.87 (Mitra and Narurkar, 1986). The degree of ionization of weak acids can be estimated using the Henderson-Hasselbalch equation. Based on this, the ionization percentage of azathioprine ranged from 0.84% at pH 5.8%–25.31% at pH 7.4. This trend indicates that at higher pH values, a larger fraction of azathioprine exists in its ionized form, which is less permeable through lipid membranes. Since only the non-ionized (neutral) form can cross lipid membranes via passive diffusion, a decrease in permeability at higher pH levels is expected (Avdeef, 2001). Using a threshold permeability value of 1 × 10^−6^ cm/s as a criterion for good permeability, our results indicate that azathioprine exhibits poor permeability across the PAMPA membrane at all tested pH values. As anticipated, permeability was higher at lower pH levels, where a greater proportion of azathioprine remains in its neutral form. However, despite its relatively low ionization across the tested pH range, the inherently poor permeability of azathioprine is likely attributed to its low lipophilicity (logP = 0.1) (Hansch et al., 1996).

Azathioprine permeability was significantly higher in all experimental groups containing probiotic bacteria compared to control groups without probiotics (Table 1). The most pronounced effect was observed at pH 7.4, with a 2.5-fold increase in Papp. At pH 6.5 and 5.8, the increases were 1.9-fold and 2.2-fold, respectively. Although the measured azathioprine concentrations in the acceptor compartment followed a similar trend (Figure 2), increasing by 57% at pH 7.4, 26% at pH 6.5, and 18% at pH 5.8, they do not fully reflect the magnitude of permeability changes, likely due to additional factors affecting drug accumulation and metabolism. The enhanced permeability can be attributed to the metabolic activity of probiotic bacteria, particularly their production of short-chain fatty acids, which lowers the local pH. As a result, the proportion of the non-ionized molecular form of azathioprine increases, facilitating passive diffusion across lipid membranes. Based on this mechanism, several studies have emphasized the role of butyrate, a key microbial metabolite, in modulating drug bioavailability by decreasing luminal pH (Ballan et al., 2020; Abbasi et al., 2020) and contributing to therapeutic outcomes. A comprehensive longitudinal study in IBD patients with integrated *in-silico* simulations demonstrated that particularly remission patients treated with azathioprine showed increased butyrate synthesis rate at the baseline (Effenberger et al., 2021). These findings indicate that microbial metabolic capacity to synthesize short-chain fatty acids is strongly associated with therapy remission in IBD patients. In accordance with these results, various previous studies concerning biologic therapy showed that an increase in butyrate production was associated with clinical remission in response to anti-TNF and anti-α4β7 integrin antibody therapy (Paramsothy et al., 2019; Aden et al., 2019). Additionally, a recently published study demonstrated that antibiotic administration, which disrupts gut microbiota, reduces the oral bioavailability of 6-TGN, highlighting the role of intestinal bacteria in thiopurine drug absorption (Wang et al., 2022). However, the precise mechanisms underlying this causality remain unclear, with pH reduction being one possible contributing factor.

Beyond modulating azathioprine permeability, probiotic bacteria significantly affected its overall mass balance, leading to a notable reduction in the total detectable amount of the drug. Namely, in the donor compartment, where azathioprine was incubated with probiotics, its concentration decreased by up to 45% after 6 h of incubation compared to the initial concentration. However, this loss was not reflected in a proportional increase in the acceptor compartment, where azathioprine levels rose by less than 1%. Accordingly, across all tested pH values, a reduction in the total mass of azathioprine (sum of donor and acceptor compartments) was observed in probiotic-treated groups, amounting to 47% at pH 5.8, 30% at pH 6.5, and 35% at pH 7.4 relative to the control group. Similar results were obtained in our previously published study using the PAMPA system with gliclazide, where probiotic bacteria exhibited a similar effect on the drug’s permeability, increasing permeability and decreasing the overall mass of the drug (Đanić et al., 2021).

These findings suggest that a portion of azathioprine may be metabolized by probiotic bacterial enzymes, aligning with previous research indicating that intestinal bacteria are able to metabolize thiopurine drugs. Notably, certain bacterial species are thought to play a key role in the complete metabolic pathway of 6-mercaptopurine (6-MP) (Lazarević et al., 2022). In addition to metabolism, probiotic bacteria may also affect azathioprine levels through bioaccumulation within the bacterial cells. Previous studies have demonstrated that some bacterial species can sequester specific drugs (Klünemann et al., 2021; Đanić et al., 2023; Ðanić et al., 2019), raising the possibility that a portion of azathioprine was taken up by bacteria, potentially representing the primary route of drug removal in this system. Drugs can enter bacterial cells not only *via* passive diffusion but also through specialized drug transport systems analogous to human transporters. These bacterial transport mechanisms may influence drug disposition and therapeutic efficacy and could even contribute to drugs resistance mechanisms (Djanic et al., 2016).

Examining the impact of DC in the PAMPA system, the observed decrease in azathioprine permeability in the presence of submicellar concentrations of DC can be attributed to complex formation between the drug and bile acid. Molecular modeling revealed that the azathioprine-DCA complex has a lower total energy than the sum of its individual components, indicating that complex formation stabilizes the system and reduces the concentration of free drug available for membrane diffusion. The primary forces contributing to the stabilization of the complex include non-1,4 van VDW interactions and attractive dipole-dipole forces. Non-1,4 VDW interactions between the steroid rings of a bile acid and the purine ring of azathioprine are likely to occur, considering their close proximity and aligning face-to-face (π–π-like interactions), leading to favorable dispersion interactions. These atoms can engage in London dispersion forces, especially between C-H groups on the steroid and aromatic carbons on the purine. Besides, dipole-dipole interactions between the hydroxyl group of a bile acid and the nitro group of azathioprine are also likely to occur considering their close proximity and proper orientation. Both hydroxyl group on a bile acid and nitro group of azathioprine are strongly polar, with a partial negative charge on the oxygen and partial positive charge of nitrogen due to electron-withdrawing effects and resonance. In some orientations, this interaction might be also a weak hydrogen bond, if the hydroxyl group donates a hydrogen to one of the nitro oxygens.

Furthermore, at physiological pH, the carboxyl group of DCA remains ionized and is oriented outward within the complex, increasing its overall hydrophilicity. This reduces the complex’s affinity for the lipid membrane, further limiting azathioprine permeability. Consistent with our findings, a study investigating gliclazide incubation with DCA demonstrated that this bile acid reduces gliclazide permeability through the PAMPA membrane, which was similarly attributed to the formation of a more hydrophilic and stable complex compared to the free drug, further supporting our hypothesis (Đanić et al., 2021). Similarly, in the case of simvastatin, drug complexation with bile acids has been shown to reduce the partition coefficient, thereby decreasing drug affinity for the lipid phase in the octanol/buffer system, which is commonly used as a model for assessing passive drug diffusion across biological membranes (Đanić et al., 2016).

Beyond affecting passive diffusion, bile acids have been shown to regulate the expression and function of various proteins and membrane transporters in both the intestinal tract and intestinal bacteria (Pavlović et al., 2018; Djanic et al., 2016; Stojančević et al., 2013; Larabi et al., 2023). These effects may further impact drug transport into bacterial cells and through intestinal wall, thereby influencing overall azathioprine absorption in the intestines. Supporting this notion, Enright et al. (Enright et al., 2018) demonstrated that bile acids can modulate the expression and activity of drug transporters in bacteria, such as P-gp. This modulation occurs through transcriptional regulation and inhibition of P-gp, leading to increased intracellular accumulation of its substrates.

The predictions provided by pkCSM and ADMETsar software tools offer valuable insights into the potential interactions between azathioprine and DC with P-gp, which could further influence their intestinal absorption and bioavailability. Based on the results of *in silico* analysis, it is unlikely that azathioprine acts as a P-gp inhibitor. This can be attributed to the fact that azathioprine does not possess the structural characteristics commonly associated with P-gp inhibitors, such as a large molecular size or specific functional groups that facilitate binding to the transporter (Prachayasittikul et al., 2017; Wang et al., 2003). However, its potential as a P-gp substrate cannot be completely ruled out. Azathioprine shares some structural similarities with known P-gp substrates, including cyclosporine and digoxin, both of which are lipophilic and contain functional groups conducive to binding with P-gp (Wang et al., 2003; Didziapetris et al., 2003). Nevertheless, azathioprine’s molecular weight of 277.4 g/mol is lower than that of typical P-gp substrates (usually greater than 400 Da), which could reduce its likelihood of being a P-gp substrate (Wang et al., 2003; Finch and Pillans, 2014). Furthermore, DC was identified as likely to be an inhibitor of P-gp. These findings suggest that co-administration of DC could enhance the intestinal absorption of azathioprine by inhibiting P-gp, potentially leading to increased bioavailability and therapeutic effects, but also raising the risk of toxicity. To further confirm these predictions, additional experimental studies, such as *in vitro* transport assays using P-gp-expressing cell lines, are necessary to determine whether azathioprine interacts with P-gp as a substrate or inhibitor and to validate these *in silico* findings.

Although the effect was not statistically significant in all groups, the degree of reduction in permeability was greater with increasing DC concentrations, as higher DC concentrations resulted in lower drug concentrations in the acceptor compartment. This effect can likely be attributed to the to the greater extent of AZA/DCA complex formation at higher DC concentrations, which decreases the pool of free azathioprine available for passive diffusion through the PAMPA membrane.

The limitations of this study primarily stem from the reliance on the *in vitro* PAMPA system, which predominantly predicts passive diffusion across artificial lipid membranes. While this model provides valuable insights into passive permeability, it does not fully capture the complexity of active transport mechanisms observed *in vivo*. To address this limitation, *in silico* analysis was incorporated to hypothesize the potential role of active transporters in the interactions between azathioprine, probiotics, and DC. However, these findings require further validation through additional experimental studies. Therefore, further *in vitro* and *in vivo* investigations are necessary to confirm these results and explore the broader implications of probiotic bacteria and bile acids on drug pharmacokinetics and therapeutic efficacy.

## 5 Conclusion

By integrating experimental findings with *in silico* analyses, this research offers new perspectives on the intricate interactions between probiotic bacteria, bile acids, and drug pharmacokinetics, providing valuable insights into pharmacomicrobiomics and personalized therapy optimization.

The study’s findings confirm that while azathioprine generally exhibits low permeability, its permeability is notably increased under more acidic conditions. Furthermore, probiotic bacteria significantly influence the overall mass balance of azathioprine in the PAMPA system, leading to a notable reduction in the total detectable amount of the drug. Despite this decrease in total drug mass, probiotic bacteria simultaneously enhance the permeability of azathioprine through the PAMPA membrane.

Our results indicate that the simultaneous administration of azathioprine and probiotics may potentially lead to increased intestinal permeability and elevated systemic concentrations of azathioprine. Additionally, there is a possibility of drug bioaccumulation and biotransformation by bacterial enzymes. This observation suggests a pharmacokinetic interaction that could contribute to the variability in therapeutic response commonly observed among IBD patients receiving thiopurine therapy. While enhanced drug levels may be beneficial in terms of efficacy for some individuals, they may also pose a risk for dose-dependent adverse effects, particularly in the absence of adequate therapeutic monitoring.

Given the widespread use of probiotics as adjunctive therapy in IBD, these findings underline the importance of carefully evaluating the timing and mode of co-administration with immunomodulatory drugs. We therefore propose that temporal separation between probiotic and azathioprine intake might reduce the risk of unintended interactions and support more consistent pharmacological effects. Additionally, our findings open a new perspective, suggesting that targeted modulation of the gut microbiota through probiotic administration may contribute to a more uniform and predictable therapeutic response. Thus, the observed impact of probiotics on azathioprine permeability provides a compelling rationale for future studies aimed at optimizing treatment strategies through personalized microbiome-based approaches.

Furthermore, the bile salt DC has been shown to reduce azathioprine permeability in a concentration-dependent manner, presumably by forming a more stable complex. *In silico* predictions suggest that while azathioprine is unlikely to act as a P-gp inhibitor, it may still interact with P-gp as a substrate, given its structural similarities to known P-gp substrates. Furthermore, the identified P-gp inhibitory potential of DC indicates that co-administration of DC with azathioprine could enhance its intestinal absorption and bioavailability, potentially improving therapeutic effects but also increasing the risk of toxicity.

Additional *in vitro* and *in vivo* studies are needed to further explore the interactions of azathioprine with gut microbiota and bile acids. Such investigations will deepen our understanding of how intestinal and probiotic bacteria, along with bile acids, influence the metabolism, pharmacokinetics, and bioavailability of azathioprine, and will provide a foundation for optimizing thiopurine-based therapy in patients with IBD.

## Data Availability

The raw data supporting the conclusions of this article will be made available by the authors, without undue reservation.
